# A Tailored mHealth Intervention for Improving Antenatal Care Seeking and Its Determinants Among Pregnant Adolescent Girls and Young Women in South Africa: Pilot Randomized Controlled Trial

**DOI:** 10.2196/59144

**Published:** 2025-10-03

**Authors:** Ronel Sewpaul, Ken Resnicow, Rik Crutzen, Natisha Dukhi, Priscilla Reddy

**Affiliations:** 1 Department of Health Promotion Care and Public Health Research Institute (CAPHRI) Maastricht University Maastricht The Netherlands; 2 Public Health, Societies and Belonging Human Sciences Research Council Pretoria South Africa; 3 School of Public Health University of Minnesota Minneapolis, MN United States; 4 Business School Imperial College London London United Kingdom

**Keywords:** antenatal care, adolescent girls and young women, adolescent pregnancy, mobile health, mHealth, tailoring, motivational interviewing, South Africa, mobile phone, adolescent, teens, youth, young women, pregnancy, pregnant adolescent, low income country, middle income country, antenatal, pilot randomized controlled trial, design, user acceptability, psychosocial

## Abstract

**Background:**

Adolescent pregnancy is of public health concern due to high rates of pregnancy-related complications and lower antenatal attendance among adolescent girls and young women. Mobile health (mHealth) interventions have the potential to improve pregnancy health behaviors and thereby birth outcomes.

**Objective:**

This pilot randomized controlled trial with pre-post design evaluated user acceptability and preliminary efficacy of an mHealth intervention to improve antenatal appointment attendance and its determinants among pregnant adolescent girls and young women in South Africa.

**Methods:**

The “Teen MomConnect” intervention entailed both fixed and 2-way tailored SMS text messages about antenatal appointment keeping and pregnancy health behaviors. The intervention content and functionality were adapted from MomConnect, a national mHealth program that sends fixed SMS text messages to pregnant women in South Africa. Pregnant adolescent girls and young women aged 13-20 years were recruited from health facilities and community networks in Cape Town during May-December 2018. Simple 1:1 randomization was used to allocate participants into the control group that received the standard MomConnect maternal health messages or the experimental group that received the Teen MomConnect intervention. A subset of experimental group participants received an in-person motivational interviewing session. Questionnaires were administered at baseline and after the end of the participants’ pregnancies. Appointment attendance data were obtained from clinic records. ANOVA, ANCOVA, and logistic regression models assessed the differences in appointments attended, awareness of HIV status, and the psychosocial determinants of antenatal attendance between the control and experimental groups.

**Results:**

Overall, 412 adolescent girls and young women were enrolled, of which 254 (62%) completed the posttest survey (64% control, 59% intervention). Patient record data were obtained for 222 of the 412 (54%; in both control and intervention) participants. A total of 84% (63/75) and 72% (54/75) rated the intervention messages highly regarding their content value and their motivational nature for behavior change, respectively. Participants responded to an average of 20% of the 2-way messages they received. Mean appointment attendance did not differ significantly between the experimental (4.86, SD 1.76) and control (4.79, SD 1.74; *P*=.79) groups. Appointment attendance was higher among intervention participants who responded to ≥50% of messages (“high-responders”; 5.08, SD 1.66) than intervention participants who responded to fewer messages (4.82, SD 1.79) and control participants (4.79, SD 1.74; *P*=.86). The mean increase in knowledge scores was significantly higher among experimental group high-responders (2.1, SD 3.17) than the control group (0.7, SD 2.73; β=1.50; *P*=.045).

**Conclusions:**

Engagement with the intervention’s 2-way messaging was low, which could have impacted the outcomes. However, the intervention content was deemed acceptable. Appointment attendance did not vary significantly between the intervention and control groups. More intensive intervention may be needed to impact appointment adherence.

**Trial Registration:**

Pan African Clinical Trial Registry (PACTR) PACTR201912734889796; https://pactr.samrc.ac.za/TrialDisplay.aspx?TrialID=9565

**International Registered Report Identifier (IRRID):**

RR2-10.2196/43654

## Introduction

South Africa has a high prevalence of pregnancy among adolescent girls and young women. The South African Demographic and Health Survey in 2016 found that 27.8% of 19-year-olds had begun childbearing [1]. This is of public health concern due to the higher risk of pregnancy complications and maternal and infant mortality for adolescent girls and young women compared to older adult pregnant women [2-4]. Antenatal care (ANC) and healthy behaviors during pregnancy are vital for healthy pregnancy outcomes. Early and routine attendance of ANC facilitates prevention, detection, management of risk factors and pregnancy complications, and the delivery of pregnancy health education [5]. However, adolescent girls and young women in South Africa generally have lower ANC attendance, and they book in later along their pregnancy for their first antenatal visit than older pregnant women [6,7]. Almost a quarter of pregnant adolescents do not attend at least four antenatal visits [8].

There is therefore a need for behavioral interventions to improve antenatal attendance and health behaviors during pregnancy among adolescent girls and young women. Mobile health (mHealth) behavior change interventions are on the rise and have the power to reach large numbers of people [9,10]. Previous mHealth interventions using SMS text messages have increased ANC uptake among pregnant women in low- and middle-income countries [11]. The South African National Department of Health’s MomConnect program [12] is one of the largest mHealth interventions in the world, having cumulatively reached over 1.7 million pregnant women between 2014 and 2017 [13]. Pregnant women are routinely registered on MomConnect when they attend ANC at public health facilities throughout South Africa. They then receive SMS text messages with maternal health information during various stages of their pregnancy, up to 1 year post birth. The messages were founded upon maternal health SMS text messages in the Mobile Alliance for Maternal Action in South Africa, which were previously shown to improve antenatal attendance in adult women [14]. MomConnect has successfully registered 63% of women attending their first antenatal appointment in South Africa [13].

The MomConnect messages are designed more for adult pregnant women, and opt-out rates of MomConnect are higher for women younger than 25 years of age [15]. We recently developed a mHealth intervention targeted specifically for pregnant adolescent girls and young women that aimed to improve antenatal attendance and its determinants, as well as health behaviors during pregnancy. The intervention, titled Teen MomConnect, included age-appropriate, tailored motivational behavior-change and reinforcement messages with iterative two-way message feedback on behaviors. Teen MomConnect was embedded in an SMS text messaging–based system similar to MomConnect. The Teen MomConnect intervention was also designed to include a motivational interviewing (MI) counseling session delivered by a trained research assistant. The development and description of the intervention and the protocol for a pilot two-arm randomized controlled trial (RCT) to evaluate the intervention can be found elsewhere [16]. The aim of this paper is to evaluate the user acceptability of the Teen MomConnect intervention and to assess its preliminary efficacy on the primary outcome of antenatal attendance. Secondary outcomes reported are awareness of HIV status, tobacco and alcohol use, and the psychosocial determinants of antenatal attendance, namely knowledge, risk perceptions, attitudes, and self-efficacy regarding antenatal attendance.

## Methods

### Study Design

We conducted a pilot 2-arm RCT with a pre-post design (registered at the Pan African Clinical Trial Registry with trial number PACTR201912734889796). The study follows the CONSORT (Consolidated Standards of Reporting Trials) statement on randomized pilot and feasibility trials (Multimedia Appendix 1) [17]. Participants were randomized into an experimental and a usual care control condition, where the experimental condition received the new Teen MomConnect intervention while the control condition received the standard MomConnect mobile SMS text messaging. The study was conducted in Cape Town, South Africa, from April 2018 to December 2019. Participants completed a posttest questionnaire after the end of their pregnancy, which included similar items to the baseline questionnaire and additional items on user acceptability. Patient records of the participants were retrieved from the health facilities after the end of their pregnancy.

### Participants and Procedure

ANC services are available free of charge at primary health care facilities within South Africa’s public health care system, which serves over 83% of the population [18]. Pregnant adolescent girls and young women aged 13-20 years of all gestational ages and who had access to a mobile phone were recruited to participate from a list of priority clinics and surrounding communities in Cape Town, South Africa. Discussions with the Western Cape Provincial Department of Health led to the identification of 16 facilities that provide ANC, where the number of pregnancies among adolescent girls and young women in the surrounding communities was highest, and the socioeconomic circumstances in those areas were challenging. Participants were approached and invited to participate while attending ANC at these facilities. Furthermore, participant recruitment was conducted in the surrounding communities by word of mouth. Participants were excluded from enrollment in the study if any one or more of their estimated date of delivery (EDD), language, name, age, and mobile number were missing or if they were already registered on the original version of MomConnect.

The initial inclusion criteria were participants up to 24 weeks pregnant. However, substantial numbers of pregnant adolescent girls and young women were more than 24 weeks pregnant at the time of being approached during recruitment. The inclusion criteria were therefore amended to include pregnant adolescent girls and young women of all gestational ages. Participant recruitment was conducted from May to December 2018. For further details on the recruitment procedure, see the study protocol [16].

A sample of 200 (100 participants per group) was targeted. It was determined that this sample size would allow us to detect a difference of 0.52 visits between the treatment groups at posttest measurement, that is, a mean of 4.1 visits in the intervention group and 3.58 visits in the control group with a pooled SD of 1.3 visits. This translated to a Cohen *d* effect size of at least 0.40 (0.52/1.3). Sample calculations applied 80% power and a type I error of 5%. We anticipated large dropout rates as observed in many adolescent studies. Given the fact that a large number of prospective participants would be expected to have missing contact details and pregnancy characteristics or to be already registered on MomConnect and would therefore be excluded from registration onto the mobile intervention, it was decided to recruit and screen three times as many participants for the baseline survey.

Recruited participants completed a consent form (for participants aged 18 years or older) or assent form (for participants younger than 18 years of age) before being enrolled into the study. In addition, parent consent forms were completed for participants aged younger than 18 years. Consenting individuals completed a self-administered baseline questionnaire using an electronic tablet or mobile phone provided by the research assistants.

Information on demographic characteristics, name, mobile phone number, age, EDD, estimated last menstrual date (LMD), and current enrollment on MomConnect was extracted from the baseline questionnaire. This information was used to determine the individuals who could be enrolled in the study. Participants were asked for the number of their own mobile phone or, alternatively, the number of a mobile phone they shared or which they could use to access the messages sent during the study. User registration to receive the MomConnect and Teen MomConnect messages required information on the user’s name, mobile phone number, language, and EDD. The EDD was necessary for the participants to receive messages tailored to the pregnancy week. In cases where the EDD was missing, an estimate was determined using the LMD. When both dates were missing, the participant was contacted telephonically numerous times to find either their EDD or LMD. Participants indicated their preferred language for their messages. The language choices were English, Afrikaans, and isiXhosa, which are the three primary languages spoken in the Western Cape province.

We used simple 1:1 randomization to allocate enrolled participants to the experimental group that received the Teen MomConnect intervention and the control group that received the standard MomConnect messages. Generation of the random allocation sequence and allocation of participants into intervention or control groups were performed by a statistician on the research team. The study was blinded for the participants because they were not told which group they were allocated to. We registered participants into the Teen MomConnect and MomConnect messaging programs in weekly or biweekly recruitment batches. Toward the end of study enrollment, we needed to randomize participants unequally to the control group to correct for a minor mistake in the randomization schema. There was a delay in turning off the unequal randomization, which resulted in a slightly uneven number of participants by treatment group at the end of study enrollment.

Participants were contacted telephonically during September-December 2018 to schedule a face-to-face MI session. Completing MI sessions between enrollment and the end of pregnancy proved to be challenging as some participants did not answer their phones after multiple calls, some failed to attend their scheduled sessions, many could not make time to attend a session due to school or other commitments, and there were difficulties with grouping participants into sessions at times and days when they were available. We therefore decided to conduct MI sessions until a third of the participants had received an MI session. The subset of MI participants was thus quota-sample driven. This would facilitate a subgroup analysis of participants within the intervention group who completed and did not complete MI, although this was not by random allocation.

After each participant’s EDD had passed, they were programmed to receive the standard MomConnect postpartum messages about caring for the newborn and postpartum maternal health. The research assistants contacted participants from a week after their EDD had passed to schedule administration of the posttest questionnaire. The posttest questionnaire administration followed the same procedure as that of the baseline. To encourage participation, incentives were provided of $3 for completing each of the baseline and posttest questionnaires and $6 for attending an MI session. At the time of writing the paper, the conversion rate was 16.7 South African Rand to US $1.

We also visited the facilities where the participants were receiving ANC to retrieve information from their patient folders. The folders contained a maternity case booklet and related information sheets that contained data from the booking appointment and subsequent antenatal appointments, as well as information recorded about the birth outcomes. The dates of the appointments attended were summed to calculate the total number of appointments attended. Maternal health care workers usually record this information in the patient records during the antenatal appointments and during and after labor.

### Intervention

The development of the intervention content was informed by formative qualitative research with pregnant adolescents about their experiences with MomConnect and with antenatal attendance, reviews of maternal health education resources, the literature on factors associated with health care seeking in pregnant adolescent girls and young women, a review of the existing MomConnect messages, and various stakeholder consultative discussions. More details about the development of the intervention can be found elsewhere [16]. Both intervention and control messaging programs used an SMS text messaging platform where participants were registered using Unstructured Supplementary Service Data [15]. An SMS text messaging platform was chosen for Teen MomConnect to facilitate comparison with MomConnect, which, at the time of intervention development in 2016, used an SMS text messaging platform. Discussions with the Department of Health revealed that SMS text messaging was preferred over a mobile app at the time because SMS text messaging did not require access to smartphones, and users would not incur data or other costs from sending and receiving SMS text messages.

The Teen MomConnect intervention content comprised an SMS text message library of 66 messages that covered 13 behavioral domains, as well as an MI counseling session. The domains were determined as being relevant to pregnancy among adolescent girls and young women in the South African context and were: appointment keeping, attitudes toward pregnancy, information about clinic attendance, general health information, alcohol, illegal drugs, smoking, nutrition, self-care and hygiene, social stigma regarding adolescent pregnancy, social support, sexually transmitted diseases, and tuberculosis. The number of messages received per participant was dependent on their pregnancy week, join week, and their responses to the tailored questions. The length of messages was restricted to 160 characters, consistent with the maximum length of an SMS text message.

The message library included 48 fixed messages and 18 two-way tailored SMS text message sets. The fixed messages did not elicit a response from the participant, while the two-way messages asked the participant to respond to a closed-ended question. The participant could respond to the question by sending back an SMS text message with the letter corresponding to their response option. The participant then received a feedback message that was tailored to their response. A customized response was also sent when the participant did not respond to the message. Participants who had missed messages from earlier pregnancy weeks were sent relevant “catch-up” messages of the ones they had missed. The language was motivational and was designed to reinforce behaviors. The development of messages was grounded in self-determination theory [19] and was based on techniques for tailoring health promotion messages used in previous studies [20-22].

There were layered, two-way tailored message sets on appointment keeping that were repeated every 4 weeks. Therefore, if a participant joined the intervention at 14 weeks pregnant, for example, they would receive six sets of appointment-keeping messages over 22 weeks. The appointment-keeping message set included asking about whether the participant knew when their next appointment was, tailored appointment reminders every 4 weeks for those who did and did not know their appointment dates, checkups of whether the appointments were actually attended, asking about the reasons for not attending their last appointment, and tailored feedback addressing various reasons for not attending each appointment. In addition, there were five fixed messages on information about appointment attendance, and the messages from other domains, like substance use, general health information, nutrition, self-care, social stigma, sexually transmitted diseases, and tuberculosis, also included content that emphasized seeking further advice, treatment, or testing from a clinic.

Messages asking about smoking, alcohol, and illegal drug use were sent at enrollment, and participants who reported past-month use of these substances were sent tailored messages every 4 weeks to follow up on the status of their substance use and motivate reduced use for those using. Examples of messages and their logic flows and timings are detailed in the study protocol [16].

A single face-to-face MI counseling session was administered by a trained research assistant during a participant’s pregnancy. MI is a style of counseling where the participant reflects on reasons for and against changing their behavior, how their behavior aligns with their goals and values, and opportunities for changing their behavior [23,24]. Research assistants underwent a 9-hour training workshop in MI from an expert MI counselor, which covered the skills of open-ended questions, positive affirmations, reflective listening, and summarizing. During the training, the research assistants role-played an MI session to practice their skills, and they were assessed and critiqued by the expert trainer. The main content areas covered in the participants’ MI sessions included appointment keeping, substance use, HIV and tuberculosis testing, medication adherence, nutrition and overall health, and pregnancy behaviors. Each session was tailored to the participant’s specific behaviors and questions so that a session could focus more on some behaviors than others. Depending on their availability, participants could schedule a one-on-one session or a group session with up to five participants.

### Measures

#### Demographic Variables

The baseline questionnaire assessed age, race (Black African, Coloured, Other), currently attending an educational institution such as school or a tertiary college (yes, no), dwelling type (informal, formal), self-perceived cost of living (food and clothes shortage, shortage of other important things, have important basics), and having previously been pregnant (yes, no). The EDD and LMD from the baseline questionnaire were used to calculate the gestational age at baseline.

#### Baseline Psychosocial Determinants of Antenatal Attendance

The baseline questionnaire included items on the following determinants: knowledge about antenatal attendance and pregnancy, risk perceptions, social support for attending ANC; peer, family, partner, and individual participant attitudes toward antenatal attendance; and self-efficacy, intention, and action-planning to attend antenatal appointments. Knowledge statements were informed by maternity booklets. The attitude, self-efficacy, peer norm, and intention items were informed by the reasoned action approach [25], and the action planning, risk perception, and social support items were informed by the I-Change Model [26]. The knowledge items were measured where 1=correct and 0=incorrect, and the other determinant items were measured on 4- or 5-point Likert scales with responses ranging from strongly disagree to strongly agree. Previous publications provide further details on the items measured per determinant [16,27].

Exploratory principal component factor analysis was performed on the items to derive scales for each determinant. The sum score of the items was used as the scale. Multimedia Appendix 2 shows the determinant scales and their reliability information. The McDonald’s omega total is often presented as a more accurate approximation of a scale’s reliability than the Cronbach α [28]. However, both indices are used as indicators of scale structure [29].

#### Outcomes

The number of appointments attended by the end of the pregnancy was extracted from the patient records and was the primary outcome to assess preliminary efficacy. In addition to appointments attended as a continuous variable, the number of antenatal appointments attended was also recoded into a binary variable for <4 versus ≥4 to distinguish the participants who attended the World Health Organization’s (WHO’s) basic ANC recommendation of at least four appointments [30]. While the WHO increased its recommended number of ANC appointments to eight contacts in recent years [31], international reporting on ANC coverage still focuses on the 4-visit indicator [8,32], and the new guidelines were relatively new to South Africa at the time of this study. We therefore reported on the percentage attending ≥4 appointments.

Secondary outcomes were self-reported awareness of HIV status (yes or no) postpartum, self-reported postpartum alcohol use in the past month among those who reported alcohol use at baseline; self-reported postpartum tobacco smoking in the past month among those who reported tobacco smoking at baseline; changes in three knowledge scale scores between baseline and posttest; and risk perceptions, positive participant attitudes, negative participant attitudes, and self-efficacy regarding antenatal attendance at posttest after the end of the pregnancy. The three knowledge scales assessed the risks of not attending ANC; preparation for childbirth, substance use, and sexually transmitted infections; and misinformation about routine antenatal and pregnancy care, respectively. The same knowledge items were asked in both baseline and posttest questionnaires. However, the other psychosocial determinant items were asked in the past tense form in the posttest questionnaire. For example, “attending clinic appointments is important for me” at baseline was rephrased to “attending clinic appointments was important for me.” This meant that the items were not directly comparable between the two time points, and the differences in their scale scores between baseline and posttest could not be computed. The reliability information on the outcome scales (knowledge, risk perceptions, attitudes, and self-efficacy) is presented in Multimedia Appendix 2.

The trial preregistration included the number of HIV tests as a secondary outcome, and it did not include awareness of HIV status. It appeared later that the number of HIV tests administered at a facility was not straightforwardly captured in the patient records. Self-reported awareness of HIV status was also deemed a more appropriate measure of awareness of one’s health than the number of HIV tests conducted per participant. Furthermore, the trial preregistration did not list knowledge, risk perceptions, attitudes, and self-efficacy as secondary outcomes.

The feasibility outcomes were satisfaction and user acceptability of the Teen MomConnect messages and the MI counseling session, as well as the number of intervention messages responded to and self-reported message usability. A total of 21 items and 14 items assessed the SMS text messages and the MI session, respectively. The items evaluating the SMS text messages asked about understandability, appropriateness, and motivation to change behaviors, while the items evaluating the MI session asked about perceptions and feelings after attending the session and motivation to change behaviors. All the items were measured on a Likert scale from 0=strongly disagree to 4=strongly agree with each statement. Exploratory principal component factor analyses were used to determine scales for each of the SMS text message and MI evaluations (Multimedia Appendix 3). The items per scale were used to create a sum score, where higher values indicated more positive evaluations of each component. The ranges of possible scores were categorized into tertiles corresponding to low, moderate, and high scores.

### Ethical Considerations

Ethical approval for the study was obtained from the Research Ethics Committee of the Human Sciences Research Council (REC 2/17/08/16), which is aligned with the Declaration of Helsinki. In addition, permissions were granted from the Provincial Government of the Western Cape and the National Department of Health to access the public health facilities. Written informed consent was obtained from participants aged 18 years or older. Written assent and written informed parental consent were obtained for participants younger than 18 years of age. The consent forms informed participants and parents of voluntary participation, the anonymity and confidentiality of their responses, and the right to withdraw from the study at any time.

### Statistical Analysis

Descriptive statistics were used to describe the characteristics of both the full sample enrolled at baseline and the analytic sample for whom data on appointments attended were available after removal of dropouts. The differences in characteristics between the groups were assessed using 2-tailed *t* tests for continuous variables and chi-square tests for discrete variables. Dropouts included participants who had a miscarriage or abortion, who opted out of the messaging platforms within the first few days, and for whom there were technical issues with registering or receiving message content. These dropouts were excluded from the analysis for efficacy and user acceptability. Descriptive statistics were also used to characterize the control and experimental conditions for whom appointment data was available. The number of antenatal appointments attended; the differences in knowledge scores between baseline and posttest; and the risk perception, positive attitude, negative attitude, and self-efficacy scores at posttest were compared between the experimental and the control group using one-way ANOVA and ANCOVA. The covariates adjusted for were age, gestational age, race, attendance at an educational institution, and self-perceived cost of living (a proxy for socioeconomic status). These covariates were informed by the literature on sociodemographic factors associated with antenatal attendance [3,33,34]. In addition, sociodemographic variables whose distributions differed significantly between the full sample and the subsample for whom appointment attendance data were available were also included as covariates. For the ANCOVA models for risk perceptions, positive attitudes, negative attitudes, and self-efficacy at posttest, their respective baseline scores were also included as covariates in the models. The underlying regression coefficients from the ANOVA and ANCOVA models were reported with 95% CIs. Simple logistic regression and multiple logistic regression adjusting for covariates were used to compare the following outcome measures between the experimental and control group: the percentage of participants who attended at least four appointments, the percentage who were aware of their HIV status at the end of their pregnancy, and the percentages of postpartum tobacco and alcohol use during the past month among those who reported using those substances at baseline. As part of the sensitivity analyses, the ANOVA and ANCOVA analyses were performed on the primary outcome of the number of appointments attended after multiple imputation via chained equations was performed to impute missing data on the number of appointments attended.

The treatment condition was recoded into three categories: control group, experimental group high responders (responded to ≥50% of the Teen MomConnect messages), and experimental group low responders (responded to <50% of the Teen MomConnect messages), to assess the possible dose-response effect of the level of engagement with the Teen MomConnect messaging content on the outcome variables. The treatment condition was also recoded into three categories: control group, experimental group who did not receive MI counseling, and experimental group who received MI counseling, to assess the effect of the intervention with and without MI on the outcome variables. These were exploratory analyses to supplement the primary intention-to-treat analyses. The same ANOVA, ANCOVA, and logistic regression analyses for the outcome variables were then performed using each of the condition-by-level-of-engagement and the condition-by-MI variables to investigate whether participants who engaged more with the intervention messages or who received MI had higher outcome values. The level of engagement and MI was not investigated for the outcomes of postpartum tobacco smoking and alcohol use because the number of participants who reported baseline use of these substances was too small.

Descriptive statistics were used to analyze the user acceptability of the Teen MomConnect messages and MI sessions, the number of Teen MomConnect messages responded to, and self-reported message usability for the participants enrolled in the intervention.

## Results

### Participation and Attrition

**Figure 1:**
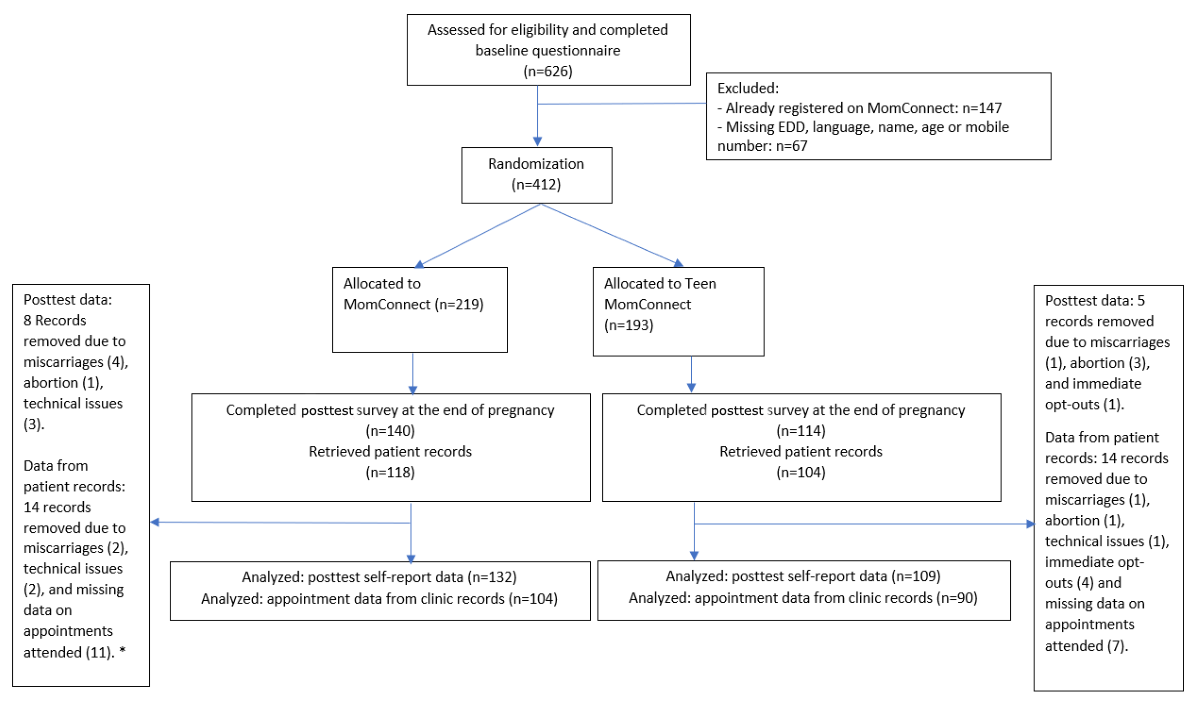
Participant flow and attrition. *Numbers do not add to 14 records because 1 participant had missing data on appointments attended and had a miscarriage. EDD: estimated date of delivery.

Figure 1 illustrates the participant flow. Recruitment occurred during May-December 2018, and posttest surveys were administered postpartum during October 2018-July 2019. A total of 626 adolescent girls and young women aged 13-20 years were assessed for eligibility and completed the baseline survey. Of these adolescent girls and young women, 412 participants were enrolled in the study, where 219 were randomized into MomConnect and 193 into Teen MomConnect. After the end of the pregnancy, 254 (62%) completed the posttest questionnaire (140/219, 64% MomConnect and 114/193, 59% Teen MomConnect). Patient records were successfully reviewed for 222 (54%) of the 412 enrolled participants (118/219, 54% MomConnect and 104/193, 54% Teen MomConnect). After removing records of participants who reported a miscarriage or abortion, who opted out of the Teen MomConnect service within the first week, and participants with technical issues later discovered with registering onto the messaging platforms, posttest data were analyzed for 241 participants (132/219, 60% MomConnect and 109/193, 56% Teen MomConnect). Furthermore, after removing records with incomplete data on appointments attended in the patient records, data on appointment attendance were analyzed for 194 participants (104/219, 47% MomConnect and 90/193, 47% Teen MomConnect).

Table 1 shows the baseline characteristics of the full sample (n=412) and the sample for whom there were data on appointments (n=194). The sample with appointment attendance data differed significantly from the full sample for the following baseline variables: age (mean 17.8, SD 1.6 vs 18.0, SD 1.6 years), gestational age (mean 22.7, SD 7.3 vs 22.0, SD 7.4 weeks), race group (40/194, 20.6% vs 123/410, 30% for Black African race), attendance at an educational institution (80/194, 41.2% vs 149/412, 36.2%) and knowledge of the risks of not attending ANC or not practicing healthy behaviors (mean score 3.8, SD 2.5 vs 3.4, SD 2.3).

**Table 1:** Characteristics of the full baseline sample and the analytic sample with data on appointment attendance.

			Full baseline sample (n=412)		Analytic sample with data on appointments (n=194)		*P* value
Age (years), mean (SD)			18.0 (1.6)		17.8 (1.6)		.01^a^
Gestational age (weeks), mean (SD)			22.0 (7.4)		22.7 (7.3)		.086
**Race group^b^, n (%)**							.001^a^
	Black African	123 (30)		40 (20.6)			
	Coloured	285 (69.5)		153 (78.9)			
	Other	2 (0.5)		1 (0.5)			
**Attending an educational institution, n (%)**							.04^a^
	Do not attend an educational institution	263 (63.8)		114 (58.8)			
	Attend an educational institution	149 (36.2)		80 (41.2)			
**Dwelling type, n (%)**							.54
	Informal	100 (24.6)		44 (23.2)			
	Formal	307 (75.4)		146 (76.8)			
**Self-perceived cost of living, n (%)**							.82
	Food and clothes shortage	92 (22.7)		41 (21.5)			
	Shortage of other important things	150 (37)		73 (38.2)			
	Have important basics	164 (40.4)		77 (40.3)			
**Had been pregnant before, n (%)**							.31
	Yes	45 (10.9)		18 (9.3)			
	No	367 (89.1)		176 (90.7)			
Past month tobacco smoking, n (%)			162 (39.4)		80 (41.5)		.43
Past month alcohol use, n (%)			119 (29)		56 (29)		.98
Knowledge score 1^c^ (range: 0-7), mean (SD)			3.8 (2.5)		3.4 (2.3)		.001^a^
Knowledge score 2^d^ (range: 0-4), mean (SD)			3.4 (1.0)		3.3 (1.1)		.08
Knowledge score 3^e^ (range: 0-3), mean (SD)			1.2 (1.0)		1.2 (1.0)		.69
Risk perceptions (range: 5-25), mean (SD)			16.3 (2.0)		16.4 (2.1)		.41
Positive participant attitudes toward ANC^f^ attendance (range: 7-35), mean (SD)			29.7 (5.2)		29.5 (5.3)		.41
Negative participant attitude toward ANC attendance (range: 5-25), mean (SD)			9.0 (3.8)		8.7 (3.6)		.10
Social support score (range: 3-12), mean (SD)			10.2 (1.8)		10.4 (1.6)		.17
Positive peer attitudes toward ANC attendance (range: 2-8), mean (SD)			6.4 (1.1)		6.4 (1.1)		.69
Negative peer attitudes toward ANC attendance (range: 3-12), mean (SD)			8.9 (1.7)		8.9 (1.8)		.78
Positive family attitudes toward ANC attendance (range: 4-20), mean (SD)			17.1 (2.7)		17.1 (2.6)		.86
Negative family attitudes toward ANC attendance (range: 3-15), mean (SD)			6.0 (2.2)		5.8 (2.1)		.09
Positive partner attitudes toward ANC attendance (range: 3-12), mean (SD)			10.0 (1.7)		10.0 (1.6)		.79
Self-efficacy score regarding ANC attendance (range: 8-32), mean (SD)			25.8 (5.0)		25.4 (4.8)		.22
Intention to attend ANC (range: 5-20), mean (SD)			16.8 (2.7)		16.7 (2.6)		.61
Action planning to attend ANC (range: 4-16), mean (SD)			12.1 (1.6)		12.0 (1.5)		.19

^a^*P*<.05.

^b^Race was self-reported by participants. Racial categories provided in the questionnaire were according to Statistics South Africa’s standard population groups: Black African, Coloured, White, and Indian. The White and Indian categories were collapsed for analysis due to small sample sizes. Race was reported not with the intention of reifying sociocultural constructs, but rather to study ongoing health disparities across groups.

^c^Knowledge score 1: knowledge of risks of not attending antenatal care or not practicing healthy behaviors.

^d^Knowledge score 2: Knowledge about preparation for childbirth, substance use, and sexually transmitted infections.

^e^Knowledge score 3: Misconceptions about antenatal care and pregnancy behaviors.

^f^ANC: antenatal care.

### Appointment Attendance and Related Determinants

Table 2 shows the estimates of appointment attendance, HIV awareness, and the psychosocial determinants of antenatal attendance after the end of pregnancy between the control and experimental groups. It further shows the regression coefficients from the underlying ANOVA and ANCOVA models for continuous outcomes and the odds ratios from the logistic regression models for the binary outcomes.

**Table 2:** Estimates of appointment attendance, risk behaviors, and the psychosocial determinants of antenatal attendance after the end of pregnancy between the control and experimental groups.

Primary outcome	Control	Intervention	Coefficient, OR^a^ (95% CI)	Adjusted coefficient, AOR^b^ (95% CI)^c^
Number of appointments attended, mean (SD)	4.8 (1.7)	4.9 (1.8)	0.07 (–0.43 to 0.57)	0.02 (–0.50 to 0.55)
Attended at least 4 appointments, n (%)	78 (75)	69 (77)	1.10 (0.57 to 2.12)	1.00 (0.49 to 2.06)
Risk perception sum score at posttest, mean (SD)	16.0 (2.4)	15.6 (1.9)	–0.43 (–1.02 to 0.15)	–0.49 (–1.11 to 0.12)
Positive participant attitudes sum score toward ANC^d^ attendance at posttest, mean (SD)	30.0 (4.8)	29.1 (5.4)	–0.89 (–2.22 to –0.44)	–0.38 (–1.73 to 0.97)
Negative participant attitudes sum score toward ANC attendance at posttest, mean (SD)	8.4 (2.8)	9.5 (4.0)	1.01 (0.13 to 1.90)	0.48 (–0.41 to 1.37)
Self-efficacy to attend ANC sum score at posttest, mean (SD)	26.5 (4.4)	25.2 (4.4)	–1.31 (–2.54 to –0.07)	–1.30 (–2.57 to –0.03)
Change in knowledge score 1^e^ between baseline and posttest survey, mean (SD)	0.7 (2.7)	0.8 (3.2)	0.08 (–0.72 to 0.89)	0.07 (–0.75 to 0.90)
Change in knowledge score 2^f^ between baseline and posttest survey, mean (SD)	0 (1.3)	0.1 (1.3)	0.14 (–0.21 to 0.48)	0.14 (–0.22 to 0.50)
Change in knowledge score 3^g^ between baseline and posttest survey, mean (SD)	0.3 (1.2)	0.5 (1.3)	0.24 (–0.09 to 0.56)	0.23 (–0.10 to 0.56)
Self-reported awareness of HIV status at posttest, n (%)	120 (93.8)	99 (94.3)	1.10 (0.37 to 3.28)	1.33 (0.41 to 4.31)
Tobacco smoking at posttest among participants who reported tobacco smoking at baseline, n (%)	32 (65.3)	26 (65.0)	1.01 (0.42 to 2.43)	0.68 (0.25 to 1.86)
Alcohol use at posttest among participants who reported alcohol use at baseline, n (%)	8 (21.1)	11 (35.5)	0.48 (0.17 to 1.42)	0.32 (0.09 to 1.10)

^a^OR: odds ratio.

^b^AOR: adjusted odds ratio.

^c^Adjusted for age, gestational age, perceived cost of living, race group, and attendance at an educational institution. The ANCOVA models for risk perception, positive and negative participant attitudes, and self-efficacy outcomes were also adjusted for their respective baseline scores.

^d^ANC: antenatal care.

^e^Knowledge score 1: knowledge of risks of not attending antenatal care or not practicing healthy behaviors.

^f^Knowledge score 2: knowledge about preparation for childbirth, substance use, and sexually transmitted infections.

^g^Knowledge score 3: misconceptions about antenatal care and pregnancy behaviors.

The number of appointments attended did not differ significantly between the Teen MomConnect intervention group (mean 4.9, SD 1.8) and the MomConnect control group (mean 4.8, SD 1.7) in both the unadjusted (β=0.07; *P*=.79) and adjusted models (β=0.02; *P*=.93). The percentage of participants who attended at least four appointments did not differ significantly between the intervention (69/90, 77%) and control groups (78/104, 75%), nor did risk perceptions, positive participant attitudes, negative participant attitudes, increase in the knowledge scores, and awareness of HIV status. Among participants who reported baseline use of tobacco (n=89) and alcohol (n=69), past-month use of each of these substances did not differ significantly between the intervention and control groups. Self-efficacy scores to attend appointments at posttest were significantly higher in the control group than in the intervention group after adjusting for the baseline score and the other covariates.

Of the participants in the intervention group, 14.3% (n=26) responded to ≥50% of the Teen MomConnect messages and were termed as the “high-responders.” The average number of appointments attended was 5.1 (SD 1.7) among participants in the intervention group who were high responders, 4.8 (SD 1.8) among intervention group participants who responded to <50% of the Teen MomConnect messages (“low-responders”), and 4.8 (SD 1.7) among control group participants (Table 3). However, these differences were not statistically significant in the unadjusted or adjusted models. The average increase in knowledge about the risks of not attending ANC was significantly higher among participants in the intervention group who were high-responders (2.1, SD 3.2) than those in the control group (0.7, SD 2.7; β=1.50; *P*=.045). There were no significant associations between the condition-by-level-of-engagement variable and the other outcome variables after adjusting for covariates.

**Table 3:** Estimates of appointment attendance, risk behaviors, and the psychosocial determinants of antenatal attendance after the end of pregnancy between the control group, intervention group low-responders, and intervention group high-responders.

	Control	Intervention—low message response	Intervention—high message response	Unadjusted model		Adjusted model^a^	
				Coefficient, OR^b^ for intervention—low-message response (95% CI)	Coefficient, OR for intervention—high-message response (95% CI)	Coefficient, AOR^c^ for intervention—low-message response (95% CI)	Coefficient, AOR for Intervention—high-message response (95% CI)
Number of appointments attended, mean (SD)	4.8 (1.7)	4.8 (1.8)	5.1 (1.7)	0.03 (–0.49 to 0.55)	0.29 (–0.73 to 1.31)	–0.04 (–0.59 to 0.51)	0.38 (–0.65 to 1.41)
Attended at least 4 appointments, n (%)	75 (78)	59 (77)	10 (77)	1.09 (0.55 to 2.18)	1.11 (0.28 to 4.35)	0.98 (0.46 to 2.07)	1.17 (0.28 to 4.91)
Risk perception sum score at posttest, mean (SD)	16.0 (2.4)	15.5 (2.0)	15.8 (1.4)	–0.48 (–1.09 to 0.14)	–0.22 (–1.35 to 0.91)	–0.53 (–1.19 to 0.12)	–0.31 (–1.47 to 0.85)
Positive participant attitudes sum score toward ANC^d^ attendance at posttest, mean (SD)	30.0 (4.8)	29.0 (5.8)	29.4 (3.1)	–0.96 (–2.38 to 0.45)	–0.53 (–3.04 to 1.98)	–0.44 (–1.88 to 1.00)	–0.11 (–2.56 to 2.34)
Negative participant attitudes sum score toward ANC attendance at posttest, mean (SD)	8.4 (2.8)	9.4 (4.2)	9.7 (3.1)	0.95 (0.01 to 1.89)	1.29 (–0.35 to 2.94)	0.33 (–0.62 to 1.28)	1.05 (–0.54 to 2.64)
Self-efficacy to attend ANC sum score at posttest, mean (SD)	26.5 (4.4)	25.4 (4.7)	24.6 (2.1)	–1.18 (–2.48 to 0.12)	–1.96 (–4.41 to 0.48)	–1.27 (–2.60 to 0.07)	–1.49 (–4.03 to 1.05)
Change in knowledge score 1^e^ between baseline and posttest survey, mean (SD)	0.7 (2.7)	0.5 (3.2)	2.1 (3.2)	–0.2 (–1.04 to 0.64)	1.38 (–0.10 to 2.86)	–0.27 (–1.13 to 0.60)	1.5 (0.03 to 2.96)
Change in knowledge score 2^f^ between baseline and posttest survey, mean (SD)	0.0 (1.3)	0.1 (1.4)	0.3 (0.7)	0.1 (–0.27 to 0.47)	0.3 (–0.33 to 0.93)	0.09 (–0.29 to 0.48)	0.33 (–0.30 to 0.95)
Change in knowledge score 3^g^ between baseline and posttest survey, mean (SD)	0.3 (1.2)	0.5 (1.3)	0.7 (1.3)	0.19 (–0.15 to 0.54)	0.43 (–0.17 to 1.03)	0.17 (–0.18 to 0.53)	0.45 (–0.15 to 1.04)
Self-reported awareness of HIV status at posttest, n (%)	120 (93.8)	78 (93)	21 (100)	0.87 (0.29 to 2.59)	—^h^	1.02 (0.31 to 3.36)	—

^a^Adjusted for age, gestational age, perceived cost of living, race group, and attendance at an educational institution. The ANCOVA models for risk perception, participant attitudes, and self-efficacy outcomes were also adjusted for their respective baseline scores.

^b^OR: odds ratio.

^c^AOR: adjusted odds ratio.

^d^ANC: antenatal care.

^e^Knowledge score 1: knowledge of risks of not attending antenatal care or not practicing healthy behaviors.

^f^Knowledge score 2: knowledge about preparation for childbirth, substance use, and sexually transmitted infections.

^g^Knowledge score 3: misconceptions about antenatal care and pregnancy behaviors.

^h^Not applicable.

Sensitivity analyses, after multiple imputation of the number of appointments attended, showed no significant differences between the number of appointments attended between the Teen MomConnect intervention group and the control group (Multimedia Appendix 4). Furthermore, the number of appointments attended did not differ significantly by the condition-by-level-of-engagement.

A third (64/193, 33.2%) of the participants in the intervention received an MI counseling session. The average number of appointments was higher in the intervention group who received MI (5.4, SD 1.7) than the control group (4.8, SD 1.7) and the intervention group who did not receive MI (4.6, SD 1.8; Table 4). Risk perceptions were significantly lower among intervention participants who did not receive MI (15.3, SD 2.1) than control group participants (16.0, SD 2.4; β=–0.80; *P*=.04). Self-efficacy to attend appointments was significantly lower in the intervention group who received MI (24.7, SD 3.5) than in the control group (26.5, SD 4.4) after adjusting for the baseline self-efficacy score and the other covariates (β=–1.84; *P*=.03).

**Table 4:** Estimates of appointment attendance, risk behaviors, and the psychosocial determinants of antenatal attendance after the end of pregnancy between the control group, intervention group without MI^a^, and intervention group with MI.

	Control	Intervention without MI	Intervention with MI	Unadjusted model			Adjusted model^b^	
				Coefficient, OR^c^ for intervention without MI (95% CI)	Coefficient, OR for intervention with MI (95% CI)	Coefficient, AOR^d^ for intervention without MI (95% CI)		Coefficient, AOR for intervention with MI (95% CI)
Number of appointments attended, mean (SD)	4.8 (1.7)	4.6 (1.8)	5.4 (1.7)	–0.23 (–0.79 to 0.34)	0.58 (–0.11 to 1.26)	–0.17 (–0.75 to 0.41)		0.42 (–0.32 to 1.17)
Attended at least 4 appointments, n (%)	78 (75)	41 (72)	28 (85)	0.85 (0.41 to 1.77)	1.87 (0.65 to 5.33)	0.9 (0.41 to 1.95)		1.36 (0.44 to 4.25)
Risk perception sum score at posttest, mean, (SD)	16.0 (2.4)	15.3 (2.1)	15.9 (1.6)	–0.71 (–1.44 to 0.01)	–0.13 (–0.87 to 0.61)	–0.8 (–1.56 to –0.05)		–0.15 (–0.94 to 0.63)
Positive participant attitudes sum score toward ANC^e^ attendance at posttest, mean (SD)	30.0 (4.8)	28.8 (6.7)	29.5 (3.4)	–1.23 (–2.87 to 0.42)	–0.53 (–2.21 to 1.15)	–0.91 (–2.55 to 0.72)		0.21 (–1.48 to 1.91)
Negative participant attitudes sum score toward ANC attendance at posttest, mean (SD)	8.4 (2.8)	9.2 (4.4)	9.8 (3.6)	0.73 (–0.37 to 1.82)	1.32 (0.20 to 2.43)	0.24 (–0.84 to 1.32)		0.74 (–0.39 to 1.87)
Self-efficacy to attend ANC sum score at posttest, mean (SD)	26.5 (4.4)	25.6 (5.0)	24.7 (3.5)	–0.89 (–2.38 to 0.60)	–1.82 (–3.42 to –0.21)	–0.86 (–2.39 to 0.67)		–1.84 (–3.47 to –0.21)
Change in knowledge score 1^f^ between baseline and posttest survey, mean (SD)	0.7 (2.7)	0.1 (3.3)	1.4 (3.1)	–0.60 (–1.58 to 0.39)	0.76 (–0.22 to 1.75)	–0.45 (–1.45 to 0.55)		0.63 (–0.39 to 1.65)
Change in knowledge score 2^g^ between baseline and posttest survey, mean (SD)	0.0 (1.3)	0.0 (1.5)	0.2 (1.0)	0.04 (–0.39 to 0.47)	0.24 (–0.20 to 0.67)	0.05 (–0.39 to 0.49)		0.24 (–0.21 to 0.69)
Change in knowledge score 3^h^ between baseline and posttest survey, mean (SD)	0.3 (1.2)	0.6 (1.3)	0.5 (1.3)	0.25 (–0.15 to 0.65)	0.22 (–0.18 to 0.63)	0.32 (–0.09 to 0.72)		0.13 (–0.28 to 0.55)
Self-reported awareness of HIV status at posttest, n (%)	120 (93.8)	49 (93)	50 (96)	0.82 (0.24 to 2.84)	1.67 (0.34 to 8.13)	0.87 (0.23 to 3.30)		2.35 (0.43 to 12.70)

^a^MI: motivational interviewing.

^b^Adjusted for age, gestational age, perceived cost of living, race group, and attendance at an educational institution. The ANCOVA models for risk perception, participant attitudes, and self-efficacy outcomes were also adjusted for their respective baseline scores.

^c^OR: odds ratio.

^d^AOR: adjusted odds ratio.

^e^ANC: antenatal care.

^f^Knowledge score 1: knowledge of risks of not attending antenatal care or not practicing healthy behaviors.

^g^Knowledge score 2: knowledge about preparation for childbirth, substance use, and sexually transmitted infections.

^h^Knowledge score 3: misconceptions about antenatal care and pregnancy behaviors.

### User Acceptability of Teen MomConnect

Analysis of the messages sent and responded to on the Teen MomConnect SMS text messaging platform showed that, on average, participants in the Teen MomConnect intervention responded to 20% of the messages that asked them questions (Table 5). Over three-quarters (143/182, 78.6%) responded to less than a third of the messages that contained questions. The mean message response rate did not differ significantly by age group, gestational age, race, self-perceived cost of living, or educational attendance (*P*>.05). When asked if they were able to respond to messages, over 60% (52/86) reported that they were able to respond always or most of the time. More than half (43/81, 53%) reported that they were able to respond to messages within 6 hours of receiving them, while 16% (13/81) were only able to respond to them after a day or longer. Among those who reported taking a long time to respond to messages, the most common reasons for not responding to messages were not checking their phones all the time (40/89, 49%) and their phones not being with them all the time due to phone sharing (31/89, 38%). Overall, 59% (52/88) reported that they would not prefer the intervention to be delivered by an app and therefore preferred SMS text messaging. In total, 84% (63/75) and 72% (54/75) rated the Teen MomConnect SMS text messaging content highly in terms of the value of the message content and the motivational nature of the messages for behavior change, respectively. The majority (64/81, 79%) expressed low negative opinions of the message content. A total of 64 (33.2%) Teen MomConnect participants received an MI counseling session. Of those who participated in the posttest survey and responded to the questions evaluating the session (n=44), 84% (37/44) and 77% (34/44) rated the MI session highly in terms of positive opinions about the MI and the motivational nature of the MI for behavior change for exercise and substance use, respectively. Among all participants (control and intervention), 22.7% (41/181) reported that they were using a different mobile number from the one used at baseline. There were no recorded harms or unintended effects among participants during the study period.

**Table 5:** Evaluation of the Teen MomConnect intervention content among participants enrolled in Teen MomConnect who participated in the posttest survey.

				Value
**Were you able to reply to all the messages? n (%)**				
	Always			24 (27.9)
	Most of the time			28 (32.6)
	Sometimes			25 (29.1)
	Never			9 (10.5)
	Missing			23 (21.1)
**How soon after seeing the messages did you reply to them? n (%)**				
	Within 6 hours			43 (53.1)
	The same day			25 (30.9)
	The next day or later			13 (16.1)
	Missing			28 (25.7)
**If you usually replied to messages long after they were sent, what were the reasons that took you so long to reply?^a^ n (%)**				
	Phone is not with me all the time because I share it with someone else			31 (37.8)
	Don’t check my phone all the time, and I see the messages much later			40 (48.8)
	I see the messages, but I do not have time to reply to them			10 (12.2)
	I thought that replying to the messages would use my airtime			8 (9.8)
**Percentage (%) of messages responded to^b^**				
	Mean (SD)			20.0 (25.6)
	<33%, n (%)			143 (78.6)
	33%-66%, n (%)			24 (13.2)
	>=66%, n (%)			15 (8.2)
**Would you have preferred the messages be delivered via an app rather than SMS text messages? n (%)**				
	Yes			36 (40.9)
	No			52 (59.1)
**Is your mobile number still the same as at baseline?^c^ n (%)**				
	Yes			140 (77.4)
	No			41 (22.7)
**Evaluations of the SMS text messages**				
	**Value of the message content**			
		Sum score (range: 0-36; mean, SD)	27.3 (5.9)	
		Low (0-12), n (%)	2 (2.7)	
		Moderate (13-24), n (%)	10 (13.3)	
		High (25-36), n (%)	63 (84.0)	
		Missing, n (%)	34 (31.2)	
	**Motivational nature of messages for behavior change**			
		Sum score (range: 0-20; mean, SD)	14.5 (3.9)	
		Low (0-6), n (%)	2 (2.7)	
		Moderate (7-13), n (%)	19 (25.3)	
		High (14-20), n (%)	54 (72.0)	
		Missing, n (%)	34 (31.2)	
	**Negative opinions of the messages**			
		Sum score (range: 0-12; mean, SD)	3.0 (2.1)	
		Low (0-4), n (%)	64 (79.0)	
		Moderate (5-8), n (%)	15 (18.5)	
		High (9-12), n (%)	2 (2.5)	
		Missing, n (%)	28 (25.7)	
**Evaluations of the MI^d^ session, n (%)**				
	Attended an MI session			64 (33.2)
	**Positive opinions about MI**			
		Sum score (range: 0-20; mean, SD)	15.5 (3.2)	
		Low (0-6), n (%)	1 (2.3)	
		Moderate (7-13), n (%)	6 (13.6)	
		High (14-20), n (%)	37 (84.1)	
		Missing, n (%)	9 (17.0)	
	**Motivational nature of MI for behavior changes in substance use and exercise**			
		Sum score (range: 0-16; mean, SD)	12.3 (3.1)	
		Low (0-5), n (%)	1 (2.3)	
		Moderate (6-11), n (%)	9 (20.5)	
		High (12-16), n (%)	34 (77.3)	
		Missing, n (%)	9 (17.0)	

^a^Multiple responses possible.

^b^Percentage of messages responded to per participant=(total number of messages responded to/total number of messages received that asked a question)×100. The percentages ranged from 0% to 100% and a mean and SD were calculated for the percentage of messages responded to.

^c^Both Teen MomConnect and MomConnect participants.

^d^MI: motivational interviewing.

## Discussion

### Principal Findings

This pilot RCT investigated the user acceptability and preliminary efficacy of Teen MomConnect. The number of antenatal appointments attended did not differ significantly between Teen MomConnect and MomConnect. While higher self-efficacy regarding attending appointments at posttest was observed in the MomConnect control group, this did not translate into higher rates of appointments attended. Higher mean increases in knowledge about the risks of not attending ANC and not practicing health behaviors during pregnancy were found among Teen MomConnect participants who interacted more frequently with the 2-way messages than the control group. In addition, the inclusion of MI in the intervention resulted in a 12% higher mean number of appointments attended (a difference of 0.57 visits) compared to the control group. This was not statistically significant, which was likely attributed to the small sample size for those who received MI. The majority of participants enrolled in the intervention regarded the messages highly in terms of content and their motivational nature for behavior change. Despite this, engagement with the 2-way messages was generally low, with participants replying to an average of 20% of the messages that asked them questions.

In comparison with our findings, a 3-arm trial among pregnant women living with HIV in Kenya also found that 2-way SMS text messages did not improve appointment attendance when compared to one-way messaging and standard care [35]. The findings of no preliminary efficacy on appointment attendance in this study can be contextualized with respect to the motivational nature, tailoring, and dose of the messages, and the fidelity of the intervention components, which relied on the frequency of engagement with the messages. First, behaviors with high resistance to change and that require larger motivation and competence to change usually need more intense or interpersonal interventions [36]. Moreover, the MomConnect fixed messages were previously tested and have already been shown to improve antenatal uptake when compared to standard care. Therefore, the Teen MomConnect intervention messages might not have adequately targeted the salient constructs affecting attendance behavior to result in higher antenatal attendance rates than MomConnect; they might not have been motivating enough, and their content may not have been sufficiently targeted to adolescent girls and young women. Second, the novelty of Teen MomConnect’s two-way SMS text messages was not used as frequently as intended. This impacted intervention fidelity because the level of tailored message feedback is dependent on participants’ responses to the two-way messages. The intervention could perhaps be more effective if it could identify and tailor appointment messages to the adolescent girls and young women with high risk for low antenatal attendance, as determined by behavioral determinants. Finally, while the intervention contained more messages on appointment attendance, it also covered a range of other behavioral domains. It is unclear the dose and intensity of messages that would be required per domain to effect behavior changes. In this study, appointment reminders were based on asking participants when their next appointment was. Precise appointment date reminders that are linked to medical records have shown more promise in improving appointment attendance behaviors [35].

mHealth interventions generally experience low engagement rates [37,38], particularly among young people who become habituated to the mobile interventions and become distracted by other activities on their mobile phones [39]. It is easy to be passively involved in mHealth interventions, and there is no direct follow-up on why messages are not responded to. Intervention engagement can be defined as the perceptions and experiences with the intervention or as intervention use [40], and higher engagement is associated with intervention effectiveness [41]. In this study, participants reported high satisfaction with the message content, suggesting favorable perceptions and experiences with the intervention and high acceptability. In terms of message use, however, the number of messages responded to was very low. The small number of participants who replied frequently to the two-way messages impacted the power to detect a dose-response relationship for high versus low message engagement. Low responsiveness to bidirectional SMS text messages has also been observed in the WelTel text-based intervention for pregnant women in Kenya [42]. Younger women aged 18-24 years were twice as likely not to reply to messages than older women, but also had high satisfaction with the intervention content. Reasons for low engagement with the messages were forgetting to reply, loss of mobile phones, and message fatigue over the duration of the interventions, and when messages seemed repetitive [42]. The iterative nature of some Teen MomConnect messages could therefore have induced message fatigue and nonresponse. Regular access to a mobile phone is crucial for adherence to the intervention and engagement with the 2-way messages. A fifth of participants changed their mobile phone number during the study, which is common in South Africa, where the majority of phones are prepaid [15]. This would have affected the messages that they were exposed to and hence responded to. Furthermore, participants cited not always checking their phones and sharing phones as common reasons for not replying to messages timeously. In addition, the intervention’s program logic allowed a few hours for participants to reply to some messages in cases where multiple messages were to be sent on the same day. This could have affected whether participants responded to the messages.

This study can be used to inform engagement rates in future text-based interventions for adolescent girls and young women. Features associated with increased adolescent engagement in mHealth studies, including apps, are increased tailoring, theory-informed messages, provision of self-monitoring and feedback, message reminders, game-based elements with rewards, and provision of peer or health care provider support [39]. The Teen MomConnect intervention included these elements to varying degrees, within the feature limitations of an SMS text messaging–based platform. Approaches that other adolescent text-based interventions used to increase engagement are quizzes or polls where the user responds by answering “myth” or “fact” and then receives the correct answer, and messages with pop culture references or slang phrases [43,44]. Teen MomConnect messages could have incorporated these features in order to increase engagement, while keeping in mind the restricted length of an SMS text message that balances both engaging and educational content, and the avoidance of sending too many messages to participants. We could have also called participants regularly to remind them to respond to messages, but this might have had limited success because previous attempts to contact them for scheduling MI sessions and posttest surveys presented difficulties with unanswered calls and phone calls directed to voicemail.

Participants who received MI reviewed it favorably. However, the feasibility of MI was low in terms of difficulties in scheduling MI sessions with participants due to unanswered calls, conflicting commitments, and participants not attending their sessions. This resulted in only one MI session being able to be delivered. Some mHealth studies confirm no significant difference in health behavior outcomes between text-based interventions and text supplemented with counseling or support groups [45,46]. The 12% increase in appointment attendance with MI found in this study was not statistically significant but warrants further investigation using larger samples. It is also possible that participants with fewer barriers to attending ANC visits were more likely to be willing to attend the MI session. Furthermore, the fidelity of the MI sessions was not assessed in this study. While the research assistants were trained and tested in MI skills, their proficiency in all aspects of MI while delivering their sessions was not measured. Suboptimal proficiency among counselors following MI training has been confirmed in other studies in South Africa [47,48]. More MI sessions may also be needed to elicit change, as effective MI interventions in pregnant women typically use 2-6 sessions [49].

Higher knowledge levels about the risks of not attending ANC and practicing healthy behaviors during pregnancy were found for participants who interacted more frequently with the Teen MomConnect intervention messages than those who did not and those in the control group. Other mHealth interventions with bidirectional messaging for adolescent girls and young women in low- and middle-income countries resulted in improvements in knowledge levels about sexual and reproductive health [50,51]. Mwenda et al [46] found that bidirectional text messages improved maternal knowledge about infant care during pregnancy. Rokicki and Fink [50] also found higher knowledge in girls who interacted more with the intervention content. Hence, the Teen MomConnect intervention messages provided participants with reproductive health information that is traditionally offered by antenatal services in a convenient, confidential, cost-effective, and adolescent-responsive way. This, in turn, enhanced their prenatal knowledge, which would equip them to make better health decisions during and after pregnancy.

A strength of the study is that the intervention development was informed by qualitative and quantitative research with pregnant adolescent girls and young women and by behavioral theories. The intervention applied tailored two-way messages and was designed to incorporate MI counseling. The intervention used SMS text messaging to facilitate comparison with the pre-existing MomConnect intervention, and SMS text messaging was the preferred method by the Department of Health at the time of the study. SMS text messages have the advantage of reaching all mobile phone users at no user cost when compared to mobile apps. Consequently, almost 60% did not prefer the messages to be delivered by an app, which can be attributed to high mobile data costs and problems with speed and coverage in South Africa [52]. In terms of limitations, first, there was low engagement with the messages, and despite participation incentives, high attrition at follow-up. Similarly, high attrition rates were found in other adolescent longitudinal studies [53,54] as well as in a study testing the MomConnect messages in pregnant women [14]. The low response rate in the follow-up survey could be because the in-person follow-up surveys were conducted postpartum at a time when the participants were preoccupied with looking after their newborns [54] and would therefore have little time and interest to be interviewed. Second, follow-up data from patient records were not collected for many participants due to difficulties with accessing patient records at their facilities. Maternal case records were not always found due to the filing systems at facilities, and some patient records were relocated to another facility or tertiary hospital where the participants gave birth. The low completion of the posttest survey and the numbers for whom appointment data were available limited the final samples used for analysis. Extensions of this work would benefit from improved strategies to improve participant engagement and interaction with the two-way messages, track and reregister participants who change mobile phone numbers, lessen attrition, and find ways in which MI can be implemented more effectively in this population.

SMS text message was relevant at the time of intervention development in 2016-2017, when smartphone access and use of the internet, social media, and apps were less prevalent in low socioeconomic groups in South Africa, among which adolescent pregnancy was highest. Smartphone access, frequent use of SMS text messaging and other apps, and internet use have now become almost ubiquitous in South Africa. Therefore, instead of text messages, content can be delivered in a wide variety of modes, such as social media sources using videos and images. The theory-based tailored two-way content used in Teen MomConnect lays a foundation for future mHealth interventions for pregnant adolescent girls and young women that use more advanced modes of technology, such as interventions that are app- or web-based or that are embedded within instant messaging services like WhatsApp. In recent years, MomConnect expanded to sending messages on WhatsApp to users who prefer this, with additional features like a chatbot answering frequently asked questions and directing questions to a helpdesk [55]. Opportunities for increased engagement and more in-depth tailoring to pregnant adolescent girls and young women, however, are provided in mobile apps. There are a few mHealth apps designed for young people in South Africa [56,57]. Apps allow for features such as gamification, greater customization and personalization, entertainment education, more efficient self-monitoring and feedback, peer forums, reminders, and notifications, which are all shown to be associated with improved appeal and relevance to youth in mHealth interventions [39].

### Conclusions

The Teen MomConnect intervention was developed to send tailored 2-way text messages on antenatal attendance and pregnancy health behaviors to pregnant adolescent girls and young women. A specific intervention was developed for adolescent girls and young women because they are inherently different from older adult women in terms of their agency, decision-making, health determinants, and social and cognitive functioning. In this pilot RCT, the Teen MomConnect intervention did not have enhanced preliminary efficacy in improving antenatal attendance when compared to the national MomConnect messages, which could be attributed to low engagement with the two-way messages. However, the intervention messages had high acceptability. Participants with high engagement had higher knowledge about ANC and pregnancy risks. Future work can investigate strategies for improving engagement and retention in mHealth studies among pregnant adolescent girls and young women. Furthermore, a more nuanced approach is needed to identify and tailor messages to adolescent girls and young women with low motivation to attend antenatal appointments. This pilot study is a stepping stone for further work on sexual and reproductive mHealth interventions in adolescent girls and young women that build on the need for tailoring in young people and that also use modes of communication that go beyond SMS text messages.

## Appendix

Multimedia Appendix 1 CONSORT 2010 checklist of information to include when reporting a pilot or feasibility trial.

Multimedia Appendix 2 Determinant Scales and reliability information.

Multimedia Appendix 3 Characteristics of the analytic sample with data on appointment attendance by treatment condition.

Multimedia Appendix 4 Sensitivity analyses.

## Abbreviations

ANC: antenatal care
CONSORT: Consolidated Standards of Reporting Trials
EDD: expected date of delivery
LMD: last menstrual date
mHealth: mobile health
MI: motivational interviewing
RCT: randomized controlled trial
WHO: World Health Organization

## Data Availability

The datasets generated and analyzed during this study are available from the corresponding author upon reasonable request.

## References

[1] National Department of Health (2019). South African medical research council, and ICF, South Africa demographic and health survey 2016. Statistics South Africa.

[2] Leftwich HK, Alves MVO (2017). Adolescent pregnancy. Pediatr Clin North Am.

[3] (2007). Adolescent pregnancy—unmet needs and undone deeds. Issues in Adolescent Health and Development.

[4] Nove A, Matthews Z, Neal S, Camacho AV (2014). Maternal mortality in adolescents compared with women of other ages: evidence from 144 countries. Lancet Global Health.

[5] (2017). WHO Recommendations on Antenatal Care for a Positive Pregnancy Experience.

[6] Gumede S, Black V, Naidoo N, Chersich MF (2017). Attendance at antenatal clinics in inner-city Johannesburg, South Africa and its associations with birth outcomes: analysis of data from birth registers at three facilities. BMC Public Health.

[7] Ebonwu J, Mumbauer A, Uys M, Wainberg ML, Medina-Marino A (2018). Determinants of late antenatal care presentation in rural and peri-urban communities in South Africa: a cross-sectional study. PLoS One.

[8] (2024). Antenatal care coverage—at least four visits (%). World Health Organization.

[9] Chan KL, Chen M (2019). Effects of social media and mobile health apps on pregnancy care: meta-analysis. JMIR Mhealth Uhealth.

[10] Rathbone AL, Prescott J (2017). The use of mobile apps and SMS messaging as physical and mental health interventions: systematic review. J Med Internet Res.

[11] Wagnew F, Dessie G, Alebel A, Mulugeta H, Belay YA, Abajobir AA (2018). Does short message service improve focused antenatal care visit and skilled birth attendance? A systematic review and meta-analysis of randomized clinical trials. Reprod Health.

[12] Bateman C (2014). Using basic technology—and corporate social responsibility—to save lives. S Afr Med J.

[13] Barron P, Peter J, LeFevre AE, Sebidi J, Bekker M, Allen R, Parsons AN, Benjamin P, Pillay Y (2018). Mobile health messaging service and helpdesk for South African mothers (MomConnect): history, successes and challenges. BMJ Global Health.

[14] Coleman J, Black V, Thorson AE, Eriksen J (2020). Evaluating the effect of maternal mHealth text messages on uptake of maternal and child health care services in South Africa: a multicentre cohort intervention study. Reprod Health.

[15] LeFevre AE, Dane P, Copley CJ, Pienaar C, Parsons AN, Engelhard M, Woods D, Bekker M, Benjamin P, Pillay Y, Barron P, Seebregts CJ, Mohan D (2018). Unpacking the performance of a mobile health information messaging program for mothers (MomConnect) in South Africa:. BMJ Global Health.

[16] Sewpaul R, Resnicow K, Crutzen R, Dukhi N, Ellahebokus A, Reddy P (2023). A tailored mHealth intervention for improving antenatal care seeking and health behavioral determinants during pregnancy among adolescent girls and young women in South Africa: development and protocol for a pilot randomized controlled trial. JMIR Res Protoc.

[17] Eldridge SM, Chan CL, Campbell MJ, Bond CM, Hopewell S, Thabane L, Lancaster GA (2016). CONSORT 2010 statement: extension to randomised pilot and feasibility trials. Pilot Feasibility Stud.

[18] (2022). General household survey 2021. Stats SA.

[19] Ryan RMH, Patrick H, Deci EL, Williams GC (2008). Facilitating health behaviour change and its maintenance: interventions based on self-determination theory. Eur Psychol.

[20] Hawkins RP, Kreuter M, Resnicow K, Fishbein M, Dijkstra A (2008). Understanding tailoring in communicating about health. Health Educ Res.

[21] Resnicow K, Davis RE, Zhang G, Konkel J, Strecher VJ, Shaikh AR, Tolsma D, Calvi J, Alexander G, Anderson JP, Wiese C (2008). Tailoring a fruit and vegetable intervention on novel motivational constructs: results of a randomized study. Ann Behav Med.

[22] Robbins LB, Pfeiffer KA, Vermeesch A, Resnicow K, You Z, An L, Wesolek SM (2013). "Girls on the Move" intervention protocol for increasing physical activity among low-active underserved urban girls: a group randomized trial. BMC Public Health.

[23] Miller WR, Rollnick S (2013). Motivational Interviewing: Helping People Change.

[24] Resnicow K, Baskin M, Rahotep S, Periasamy S, Rollnick S (2011). Handbook of Motivational Counseling: Motivating People for Change.

[25] Fishbein M, Ajzen I (2010). Predicting and Changing Behavior: The Reasoned Action Approach.

[26] de Vries H (2017). An integrated approach for understanding health behavior; the I-change model as an example. PBSIJ.

[27] Sewpaul R, Crutzen R, Reddy P (2022). Psychosocial determinants of the intention and self-efficacy to attend antenatal appointments among pregnant adolescents and young women in Cape Town, South Africa: a cross-sectional study. BMC Public Health.

[28] Crutzen R, Peters GY (2017). Scale quality: alpha is an inadequate estimate and factor-analytic evidence is needed first of all. Health Psychol Rev.

[29] Peters GJY (2014). The alpha and the omega of scale reliability and validity: why and how to abandon Cronbach's alpha and the route towards more comprehensive assessment of scale quality. Euro Health Psychol.

[30] Villar J, Ba'aqeel H, Piaggio G, Lumbiganon P, Belizán JM, Farnot U, Al-Mazrou Y, Carroli G, Pinol A, Donner A, Langer A, Nigenda G, Mugford M, Fox-Rushby J, Hutton G, Bergsjø P, Bakketeig L, Berendes H, Garcia J (2001). WHO antenatal care randomised trial for the evaluation of a new model of routine antenatal care. Lancet.

[31] World Health Organization (2016). WHO Recommendations on Antenatal Care for a Positive Pregnancy Experience.

[32] United Nations Children's Fund (2021). The state of the world's children 2021. On My Mind: Promoting, Protecting and Caring for Children’s Mental Health.

[33] Khuzaiyah S, Mumin KHA, McKenna L, Hashim SH (2023). Health-seeking behaviours of pregnant adolescents: a scoping review. Br J Midwifery.

[34] Hackett K, Lenters L, Vandermorris A, LaFleur C, Newton S, Ndeki S, Zlotkin S (2019). How can engagement of adolescents in antenatal care be enhanced? Learning from the perspectives of young mothers in Ghana and Tanzania. BMC Pregnancy Childbirth.

[35] Kinuthia J, Ronen K, Unger JA, Jiang W, Matemo D, Perrier T, Osborn L, Chohan BH, Drake AL, Richardson BA, John-Stewart G (2021). SMS messaging to improve retention and viral suppression in prevention of mother-to-child HIV transmission (PMTCT) programs in Kenya: a 3-arm randomized clinical trial. PLoS Med.

[36] Resnicow K, Teixeira PJ, Williams GC (2017). Efficient allocation of public health and behavior change resources: the "Difficulty by Motivation" matrix. Am J Public Health.

[37] Jakob R, Harperink S, Rudolf AM, Fleisch E, Haug S, Mair JL, Salamanca-Sanabria A, Kowatsch T (2022). Factors influencing adherence to mHealth apps for prevention or management of noncommunicable diseases: systematic review. J Med Internet Res.

[38] Hesser H (2020). Estimating causal effects of internet interventions in the context of nonadherence. Internet Interventions.

[39] Hightow-Weidman LB, Horvath KJ, Scott H, Hill-Rorie J, Bauermeister JA (2021). Engaging youth in mHealth: what works and how can we be sure?. Mhealth.

[40] Short CE, DeSmet A, Woods C, Williams SL, Maher C, Middelweerd A, Müller AM, Wark PA, Vandelanotte C, Poppe L, Hingle MD, Crutzen R (2018). Measuring engagement in eHealth and mHealth behavior change interventions: viewpoint of methodologies. J Med Internet Res.

[41] Donkin L, Christensen H, Naismith SL, Neal B, Hickie IB, Glozier N (2011). A systematic review of the impact of adherence on the effectiveness of e-therapies. J Med Internet Res.

[42] Nordberg B, Kaguiri E, de Angeles KJC, Gabriel EE, van der Kop ML, Mwangi W, Lester RT, Were E, Ekström AM, Rautiainen S (2024). The use, adherence, and evaluation of interactive text-messaging among women admitted to prevention of mother-to-child transmission of HIV care in Kenya (WelTel PMTCT). BMC Pregnancy Childbirth.

[43] Devine S, Leeds C, Shlay JC, Leytem A, Beum R, Bull S (2015). Methods to assess youth engagement in a text messaging supplement to an effective teen pregnancy program. J Biomed Inform.

[44] Wrobel J, Silvasstar J, Peterson R, Sumbundu K, Kelley A, Stephens D, Craig Rushing S, Bull S (2022). Text messaging intervention for mental wellness in American Indian and alaska native teens and young adults (BRAVE Study): analysis of user engagement patterns. JMIR Form Res.

[45] Head KJ, Noar SM, Iannarino NT, Harrington NG (2013). Efficacy of text messaging-based interventions for health promotion: a meta-analysis. Soc Sci Med.

[46] Mwenda V, Makena I, Ogweno V, Obonyo J, Were V (2023). The effectiveness of interactive text messaging and structured psychosocial support groups on developmental milestones of children from adolescent pregnancies in Kenya: quasi-experimental study. JMIR Pediatr Parent.

[47] Mash R, Baldassini G, Mkhatshwa H, Sayeed I, Ndapeua S (2014). Reflections on the training of counsellors in motivational interviewing for programmes for the prevention of mother to child transmission of HIV in sub-Saharan Africa. S Afr Fam Pract.

[48] Dewing S, Mathews C, Cloete A, Schaay N, Shah M, Simbayi L, Louw J (2013). From research to practice: lay adherence counsellors' fidelity to an evidence-based intervention for promoting adherence to antiretroviral treatment in the Western cape, South Africa. AIDS Behav.

[49] van der Windt M, van Zundert S, Schoenmakers S, Jansen P, van Rossem L, Steegers-Theunissen R (2021). Effective psychological therapies to improve lifestyle behaviors in (pre)pregnant women: a systematic review. Prev Med Rep.

[50] Rokicki S, Fink G (2017). Assessing the reach and effectiveness of mHealth: evidence from a reproductive health program for adolescent girls in Ghana. BMC Public Health.

[51] Akande OW, Muzigaba M, Igumbor EU, Elimian K, Bolarinwa OA, Musa OI, Akande TM (2024). The effectiveness of an m-health intervention on the sexual and reproductive health of in-school adolescents: a cluster randomized controlled trial in Nigeria. Reprod Health.

[52] Wallis L, Blessing P, Dalwai M, Shin SD (2017). Integrating mHealth at point of care in low- and middle-income settings: the system perspective. Global Health Action.

[53] Laurenzi CA, du Toit S, Ameyan W, Melendez-Torres GJ, Kara T, Brand A, Chideya Y, Abrahams N, Bradshaw M, Page DT, Ford N, Sam-Agudu NA, Mark D, Vitoria M, Penazzato M, Willis N, Armstrong A, Skeen S (2021). Psychosocial interventions for improving engagement in care and health and behavioural outcomes for adolescents and young people living with HIV: a systematic review and meta-analysis. J Int AIDS Soc.

[54] Pinto-Foltz MD, Logsdon MC, Derrick A (2011). Engaging adolescent mothers in a longitudinal mental health intervention study: challenges and lessons learned. Issues Ment Health Nurs.

[55] (2023). South African programme MomConnect. Jembi Health Systems.

[56] (2021). mHealth for adolescents and young people living with HIV: best practices, tools, and case studies. Paediatric-Adolescent Treatment Africa (PATA).

[57] Mulawa MI, Mtukushe B, Knippler ET, Matiwane M, Al-Mujtaba M, Muessig KE, Hoare J, Hightow-Weidman LB (2023). Supporting adolescents with HIV in South Africa through an adherence-supporting app: mixed methods beta-testing study. JMIR Form Res.

