# Supplementary material for: A Tailored mHealth Intervention for Improving Antenatal Care Seeking and Its Determinants Among Pregnant Adolescent Girls and Young Women in South Africa: Pilot Randomized Controlled Trial
Source: JMIR Mhealth Uhealth. 2025 Oct 3;13:e59144. doi: 10.2196/59144 (PMC12534758; doi:10.2196/59144)
Supplement: Multimedia Appendix 2 [file mhealth_v13i1e59144_app2.docx]

| Determinant category and determinant scale | Number of items in scale | α | Omega total |
| --- | --- | --- | --- |
| **Baseline survey** | | | |
| **Knowledge about pregnancy and antenatal care** | | | |
| Knowledge of risks of not attending antenatal care or not practising healthy behaviors | 7 | 0.84 | 0.88 |
| Knowledge about preparation for childbirth, substance use, and sexually transmitted infections | 4 | 0.73 | 0.79 |
| Misconceptions about antenatal care and pregnancy behaviors | 3 | 0.34 | 0.37 |
| **Peer attitudes about antenatal care** | | | |
| Negative peer attitudes | 3 | 0.72 | 0.76 |
| Positive peer attitudes | 2 | 0.72 | 0.72 |
| **Family attitudes about antenatal care** | | | |
| Positive family attitudes | 4 | 0.92 | 0.94 |
| Negative family attitudes | 3 | 0.64 | 0.67 |
| **Partner attitudes about antenatal care** | | | |
| Positive partner attitudes | 3 | 0.93 | 0.94 |
| **Risk perceptions** |  |  |  |
| Risk perceptions of antenatal care and pregnancy problems | 5 | 0.78 | 0.82 |
| **Individual participant attitudes about antenatal care** | | | |
| Positive participant attitudes | 7 | 0.95 | 0.96 |
| Negative participant attitudes | 5 | 0.89 | 0.92 |
| **Self-efficacy to attend antenatal care** |  |  |  |
| Self-efficacy to attend antenatal care | 8 | 0.96 | 0.97 |
| **Intentions to attend antenatal care** |  |  |  |
| Intentions to attend antenatal care | 5 | 0.95 | 0.97 |
| **Action planning to attend antenatal care** |  |  |  |
| Action planning to attend antenatal care | 4 | 0.85 | 0.89 |
| **Posttest survey** | | | |
| **Knowledge** | | | |
| Knowledge of risks of not attending antenatal care or not practising good behaviors | 7 | 0.83 | 0.89 |
| Knowledge about preparation for childbirth, substance use, and sexually transmitted infections | 4 | 0.78 | 0.83 |
| Misconceptions about antenatal care and pregnancy behaviors | 3 | 0.44 | 0.46 |
| **Risk perceptions** |  |  |  |
| Risk perceptions of antenatal care and pregnancy problems | 5 | 0.68 | 0.74 |
| **Individual participant attitudes about antenatal care** | | | |
| Positive participant attitudes | 7 | 0.95 | 0.96 |
| Negative participant attitudes | 5 | 0.89 | 0.92 |
| Self-efficacy |  |  |  |
| Self-efficacy to attend antenatal care | 8 | 0.94 | 0.97 |
| **Evaluation of SMS text messaging intervention** | | | |
| Value of the message content | 9 | 0.91 | 0.93 |
| Motivational nature of messages to change behaviors | 5 | 0.82 | 0.85 |
| Negative perceptions of the messages | 3 | 0.67 | 0.69 |
| **Evaluation of MI^a^ session** | | | |
| Positive attitudes about MI | 5 | 0.90 | 0.95 |
| Perceptions of MI session being motivating in changing behaviors in substance use and exercise | 4 | 0.91 | 0.95 |

^a^MI: motivational interviewing.
