# Supplementary material for: A Tailored mHealth Intervention for Improving Antenatal Care Seeking and Its Determinants Among Pregnant Adolescent Girls and Young Women in South Africa: Pilot Randomized Controlled Trial
Source: JMIR Mhealth Uhealth. 2025 Oct 3;13:e59144. doi: 10.2196/59144 (PMC12534758; doi:10.2196/59144)
Supplement: Multimedia Appendix 3 [file mhealth_v13i1e59144_app3.docx]

|  | | Analytic sample with data on appointments (n=194) | |  |
| --- | --- | --- | --- | --- |
|  |  | Control (n=104) | Intervention (n=90) |  |
| Age (years), mean (SD) | | 17.6 (1.6) | 17.9 (1.5) | |
| Gestational age (weeks), mean (SD) | | 23.9 (7.4) | 21.3 (6.9) | |
| **Race group ^a^, n (%)** | | | | |
|  | Black African | 19 (18.3) | 21 (23.3) | |
|  | Coloured | 84 (80.8) | 69 (76.7) | |
|  | Other | 1 (1.0) | 0 (0.0) | |
| **Attending an educational institution, n (%)** | | | | |
|  | Do not attend educational institution | 57 (54.8) | 57 (63.3) | |
|  | Attend educational institution | 47 (45.2) | 33 (36.7) | |
| **Dwelling type, n (%)** | | | | |
|  | Informal | 18 (17.8) | 26 (29.2) | |
|  | Formal | 83 (82.2) | 63 (70.8) | |
| **Self-perceived cost of living, n (%)** | | | | |
|  | Food and clothes shortage | 15 (14.9) | 26 (28.9) | |
|  | Shortage of other important things | 35 (34.7) | 38 (42.2) | |
|  | Have important basics | 50.5 (51.0) | 26 (28.9) | |
| **Had been pregnant before, n (%)** | | | | |
|  | Yes | 10 (9.6) | 8 (8.9) | |
|  | No | 94 (90.4) | 82 (91.1) | |
|  | Past month tobacco smoking, n (%) | 43 (41.4) | 37 (41.6) | |
|  | Past month alcohol use, n (%) | 34 (32.7) | 22 (24.7) | |
|  | Knowledge score 1 (range: 0-7), mean (SD) | 3.3 (2.3) | 3.5 (2.4) | |
|  | Knowledge score 2 (range: 0-4), mean (SD) | 3.3 (1.0) | 3.3 (1.1) | |
|  | Knowledge score 3 (range: 0-3), mean (SD) | 1.1 (0.9) | 1.3 (1.0) | |
|  | Risk perceptions (range: 5-25), mean (SD) | 16.4 (2.1) | 16.4 (2.2) | |
|  | Positive participant attitudes towards ANC^b^ attendance (range: 7-35), mean (SD) | 29.4 (5.0) | 29.6 (5.7) | |
|  | Negative participant attitude towards ANC attendance (range: 5-25), mean (SD) | 8.3 (3.1) | 9.1 (4.1) | |
|  | Social support score (range: 3-12), mean (SD) | 10.3 (1.7) | 10.5 (1.5) | |
|  | Positive peer attitudes towards ANC attendance (range: 2-8), mean (SD) | 6.5 (1.0) | 6.2 (1.2) | |
|  | Negative peer attitudes towards ANC attendance (range: 3-12), mean (SD) | 9 (1.7) | 8.8 (1.9) | |
|  | Positive family attitudes towards ANC attendance (range: 4-20), mean (SD) | 17.4 (2.2) | 16.8 (3.1) | |
|  | Negative family attitudes towards ANC attendance (range: 3-15), mean (SD) | 5.7 (2.1) | 6 (2.1) | |
|  | Positive partner attitudes towards ANC attendance (range: 3-12), mean (SD) | 10.1 (1.5) | 9.9 (1.8) | |
|  | Self-efficacy score regarding ANC attendance (range: 8-32), mean (SD) | 25.5 (4.3) | 25.4 (5.3) | |
|  | Intention to attend ANC (range: 5-20), mean (SD) | 16.8 (2.6) | 16.6 (2.7) | |
|  | Action planning to attend ANC (range: 4-16), mean (SD) | 12 (1.4) | 12 (1.7) | |

^a^. Race was self-reported by participants. Racial categories provided in the questionnaire were according to Statistics South Africa’s standard population groups: Black African, Coloured, White and Indian. The White and Indian categories were collapsed for analysis due to small sample sizes. Race was reported not with the intention of reifying sociocultural constructs, but rather to study ongoing health disparities across groups.

^b^ANC: antenatal care.

Knowledge score 1: knowledge of risks of not attending antenatal care or not practising healthy behaviours, Knowledge score 2: Knowledge about preparation for

childbirth, substance use and sexually transmitted infections, Knowledge score 3: Misconceptions about antenatal care and pregnancy behaviours.
