# Supplementary material for: A Tailored mHealth Intervention for Improving Antenatal Care Seeking and Its Determinants Among Pregnant Adolescent Girls and Young Women in South Africa: Pilot Randomized Controlled Trial
Source: JMIR Mhealth Uhealth. 2025 Oct 3;13:e59144. doi: 10.2196/59144 (PMC12534758; doi:10.2196/59144)
Supplement: Multimedia Appendix 4 [file mhealth_v13i1e59144_app4.docx]

Data on the number of appointments attended was missing for 208 of the 412 participants (50.5%).

| ***Primary outcome*** | Control | Intervention | Coefficient / OR (95% CI) | Adjusted Coefficient / AOR^a^ |
| --- | --- | --- | --- | --- |
| Number of appointments attended (mean (SE)) | 4.8 (0.2) | 4.9 (0.2) | 0.07 (-0.43 - 0.57) | 0.02 (-0.50 - 0.55) |

Table S1. Estimates of appointment attendance between the control and experimental groups before multiple imputation.

OR: Odds ratio, AOR: Adjusted odds ratio, SD: standard deviation.

a. Adjusted for age, gestational age, perceived cost of living, race group and attendance at an educational institution.

Table S2. Estimates of appointment attendance between the control group, intervention group low-responders and intervention group high-responders, before multiple imputation.

|  | | | | Unadjusted model | | Adjusted model ^a^ | |
| --- | --- | --- | --- | --- | --- | --- | --- |
| ***Primary outcome*** | Control | Intervention- low message response | Intervention - high message response | Coefficient / OR for Intervention-low-message response | Coefficient / OR for Intervention-high-message response | Coefficient / AOR for Intervention-low-message response | Coefficient / AOR for Intervention-high-message response |
| Number of appointments attended (mean, SE) | 4.8 (0.2) | 4.8 (0.2) | 5.1 (0.5) | 0.03  (-0.49 - 0.55) | 0.29 (-0.73 - 1.31) | -0.04  (-0.59 - 0.51) | 0.38  (-0.65 - 1.41) |

OR: Odds ratio, AOR: Adjusted odds ratio, SD: standard deviation.

a. Adjusted for age, gestational age, perceived cost of living, race group and attendance at an educational institution.

Prior to multiple imputation, an investigation of missingness patterns and analyses to identify auxiliary variables associated with the number of appointments attended and variables associated with the missingness in appointments attended was conducted. Potential auxiliary variables investigated were the demographic and pregnancy related characteristics at baseline and the psychosocial determinant scores of antenatal care attendance at post-test and baseline. The following were identified:

- Baseline self-efficacy to attend appointments, whether the participant’s pregnancy was registered at a clinic, baseline positive peer attitudes, and knowledge of pregnancy risk factors at post-test all had strong bivariate associations with the number of appointments attended.
- Age, Gestational age, attendance at an educational institution, perceived cost of living, race, baseline risk perception score, and baseline positive participant attitudes were all associated with missingness in the number of appointments attended.

These variables together with the treatment group (Intervention/Control) were used in the multiple imputation procedure.

Multiple imputation with chained equations and 10 imputations was performed to impute the number of appointments attended.

Tables S3 and S4 show the estimates of appointment attendance after multiple imputation.

Table S3. Estimates of appointment attendance between the control and experimental groups after multiple imputation.

| ***Primary outcome*** | Control | Intervention | Coefficient / OR (95% CI) | Adjusted Coefficient / AOR^a^ |
| --- | --- | --- | --- | --- |
| Number of appointments attended (mean (SE)) | 4.8 (0.2) | 4.8 (0.2) | 0.03 (-0.42 - 0.48) | 0.05 (-0.43 - 0.53) |

OR: Odds ratio, AOR: Adjusted odds ratio, SE: standard error

Table S4. Estimates of appointment attendance between the control group, intervention group low-responders and intervention group high-responders, after multiple imputation.

|  |  |  |  | Unadjusted model | | Adjusted model ^a^ | | |  |
| --- | --- | --- | --- | --- | --- | --- | --- | --- | --- |
| ***Primary outcome*** | Control | Intervention- low message response | Intervention - high message response | Coefficient / OR for Intervention-low-message response | Coefficient / OR for Intervention-high-message response | | Coefficient / AOR for Intervention-low-message response | Coefficient / AOR for Intervention-high-message response | |
| Number of appointments attended (mean, SE) | 4.8 (0.2) | 4.8 (02) | 5.0 (0.5) | 0.01  (-0.45 - 0.46) | 0.19  (-0.75 – 1.12) | | 0.02  (-0.48 - 0.51) | 0.25  (-0.68 – 1.18) | |

OR: Odds ratio, AOR: Adjusted odds ratio, SE: standard error.
